# Trainable soft electronics with memory in liquid crystal polymers

**DOI:** 10.1126/sciadv.aee8616

**Published:** 2026-07-08

**Authors:** Pengrong Lyu, Samuël A. M. Weima, Jaeryang Baek, Ouassim L’Karkouri, Dirk J. Broer, Mert O. Astam, Danqing Liu

**Affiliations:** ^1^ Institute for Complex Molecular Systems, Eindhoven University of Technology, Den Dolech 2, 5612 AZ Eindhoven, Netherlands.; ^2^ Department of Chemical Engineering and Chemistry, Eindhoven University of Technology, Den Dolech 2, 5612 AZ Eindhoven, Netherlands.; ^3^ School of Computing Science, Simon Fraser University, Burnaby, BC, Canada.

## Abstract

State-of-the-art soft materials can be engineered as sensors and actuators, yet, methods for learning from external information remain a subject of current research. Inspired by the use of large datasets to train artificial intelligence, tuning physical responsiveness to relayed data would introduce learning behavior in soft materials. In this work, we develop a trainable liquid crystal oligomer network (LCON) that stores digital information directly into its molecular configuration. By functionalizing the anisotropic LCON with photo-switchable azobenzene, we simultaneously integrate basic logic and memory in a material through a binary-state system; we coin this design the trainable self-propelled gate (T-SPG). We can tune the memory of our T-SPG with photonic stimuli, allowing the system to be trained by a conventional digital controller. We demonstrate the trainability of the T-SPG through two hierarchical tasks: a lower-level binary classification task where the decision boundary is stored as material memory, and a higher-level motion task that uses the stored memory to trigger actuation.

## Teaser

Trainable liquid crystal polymer stores digital information in its molecular structure, enabling logic, memory, and actuation.

## INTRODUCTION

**Fig. 1.**
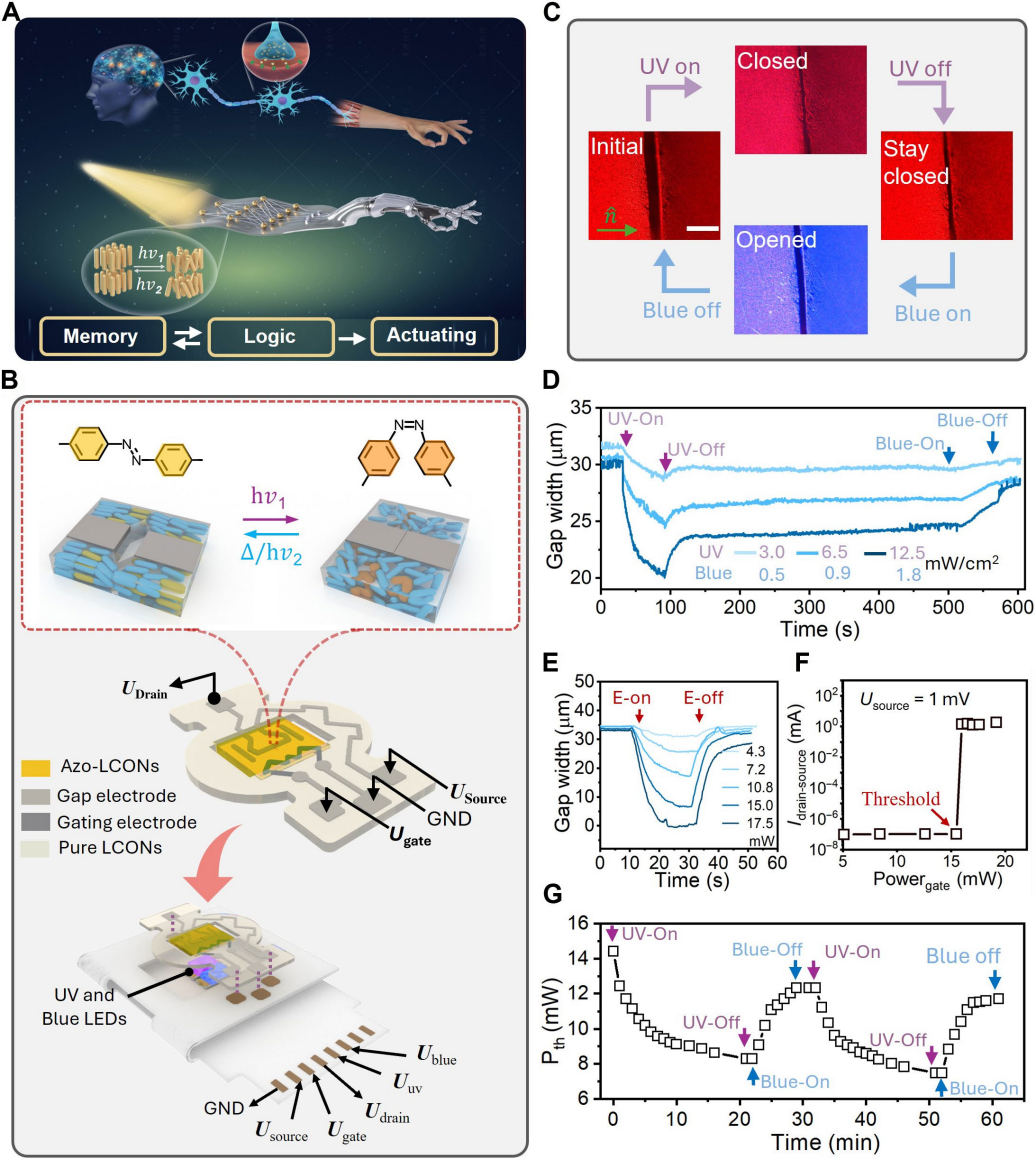
Design and working principle of the trainable LCON. (**A**) Conceptual comparison of biological systems with a soft intelligent system based on LCONs. In biological systems, the interconnected neurons process information, store memories, and coordinate actuation. Our soft intelligent system mimics the biological system. (**B**) Schematic describing the structure of the T-SPG device. From top to bottom: illustration of azobenzene isomerization under UV and blue light conditions; schematic layout of the T-SPG; assembled T-SPG integrated with a folded F-PCB, where the purple dotted lines indicate the connecting interface between the T-SPG and the F-PCB. (**C**) Optical microscopy images of the Azo-LCON switch under different light conditions: initial state in the dark; gap closing upon UV irradiation; gap closure maintained after the UV light is switched off; gap reopening upon blue light irradiation. Scale bar, 75 μm. (**D**) The influence of UV and blue light intensities on the gap width. (**E**) Gap width changes under different power. (**F**) Drain-source current (*I_drain-source_*) as a function of input power, measured after applying the *U_gate_* for 30 s to ensure thermal stability. (**G**) Reversible tuning of threshold power (*P_th_*) through alternating UV and blue light irradiation. Figure created with Cinema 4D and Adobe Photoshop.

The ability to learn and adapt is a defining feature of biological intelligence, particularly in humans, where interconnected neurons process information, store memories, and trigger actuation (Fig. 1A) (*1*–*3*). Inspired by this, artificial neural networks (ANNs) have been developed to perform complex tasks such as classification, prediction, and robotic control (*4*–*7*). ANNs learn from labeled datasets, where networks iteratively adjust internal parameters using labeled examples to minimize errors, enabling data-driven decision-making (*8*, *9*). To push the boundaries of dataset-driven learning toward hardware-integrated systems, physical neural networks (e.g., optical or mechanical computing systems) have been proposed (*10*–*12*). However, they still struggle to combine processing, memory, and actuating functionalities within a cohesive framework, unlike biological systems. One promising approach for overcoming this limitation is the use of responsive materials (*13*–*19*), such as liquid crystal polymers (*20*–*27*), which can dynamically reconfigure their molecular orientation in response to external stimuli such as light (*28*–*36*), heat (*37*–*40*), and electricity (*41*–*43*). Compared with other responsive materials [e.g., hydrogels (*44*), dielectric elastomers (*45*), shape memory polymers (*46*), and magneto-responsive materials (*47*)], liquid crystal polymers offer a unique combination of properties, including compatibility with standard electronics, programmable actuation via molecular orientation, and large reversible strain, that are essential for integrating logic, memory, and actuation into a single material platform. Yet, to integrate dataset-driven training into material intelligence, molecular transitions need to be interpretable as electrical signals (*48*–*50*).

Therefore, here, we propose a liquid crystal oligomer network (LCON) that can be trained by a digital control system, storing the results directly within its molecular configuration. Logic and memory functionalities are achieved through a binary-state system trained, which can be used to trigger actuation based on material training (Fig. 1A); we coin this the system as the trainable self-propelled gate (T-SPG; Fig. 1B). The T-SPG acts as a logic switch that is responsive to electricity for basic signal processing, which also controls the conductivity of the system; light responsiveness, obtained via incorporation of photo-responsive azobenzene, is used to store memory. This switch consists of a well-aligned LCON copolymerized with diacrylate functionalized azobenzene (Azo-LCON), with a pair of electrodes that initially disconnected with a gap; Azo-LCON is selected for its high network mobility and large responsive strain. More details about chemical composition and the properties of the Azo-LCON are provided in fig. S1.

## RESULTS

The fabrication of the Azo-LCON switch follows a previously reported method (*51*), and the resulting gap structure is shown in Fig. 1C. The initial gap width is determined by the fabrication conditions (fig. S2) and can be modulated with both light and electrothermal stimuli. First, we irradiate the gap with ultraviolet (UV) light, initiating the trans-to-cis isomerization of azobenzene to introduce a memory function. This isomerization disrupts order of the initially well-aligned mesogens (fig. S3), the alignment of which being defined as order parameter. As a result, Azo-LCON contracts along the long axis (director, $\hat{n}$), with the progressive gap closure rate being dependent on the UV light intensity (Fig. 1D and movie S1). After the UV exposure ceases, a slight rebound occurs because of the photothermal effect related to a temperature-dependent degree of alignment, which is more pronounced at higher UV intensities. When the gap width stabilizes, the memory is established. We select a commonly used azobenzene that allows the Azo-LCON switch to return to its original state under dark conditions for 10 hours (fig. S4). To erase the stored memory, we irradiate the gap with low-intensity blue light (≤1.8 mW/cm^2^), accelerating cis-to-trans isomerization, thereby restoring the order parameter and reopening the conductive gap within approximately 1 min (Fig. 1, C and D). Further increasing the blue light intensity can further accelerate the azobenzene cis-to-trans isomerization but also bring undesired heating in the polymer network, which reduces the order parameter and causes unintended gap closure. At extremely high intensities (e.g., 12 mW/cm^2^), gap reopening can even be completely inhibited (fig. S5). Thus, optimal UV and blue light intensities of ~3.0 and 1.8 mW/cm^2^, respectively, are selected for reliable memory writing and erasure.

To activate the logic function within the T-SPG, we design a gating electrode positioned near the Azo-LCON switch (Fig. 1B). When an electric signal (*U*_*gate*_) is applied, current-induced Joule heating rises the temperature, reducing the order parameter of the Azo-LCON and causes contraction along the long axis, thereby narrowing the gap (movie S2). As shown in Fig. 1E, the gap width changes under different *U*_*gate*_, demonstrating that higher power accelerates the contraction. A sharp increase in drain-source current (*I*_*drain-source*_) is observed (Fig. 1F), indicating gap closure and defining the corresponding threshold input power (*P*_*th*_), calculated as *U*_*gate*_^*2*^/*R*. To avoid electrode damage from mechanical stress upon full gap closure, all measurements were performed with input power limited to below 20 mW. In this specific experiment, the *U*_*gate*_ is a constant 6.2-V input signal and *R* is the resistance of the gating electrode (2.5 kΩ). Once the input electric signal is turned off, the Azo-LCON cools down and the gap returns to its initial width.

Furthermore, we modulate the *P*_*th*_ of the T-SPG via light irradiation. Upon UV exposure, *P*_*th*_ gradually decreases, reducing the required electrical power by approximately 40% after 20 min. To restore *P*_*th*_, blue light irradiation is used, achieving stabilization within 5 min; this process can be repeated more than 50 times (fig. S6). To use the logic and memory functions of the Azo-LCON, we develop a device as illustrated in Fig. 1B. The T-SPG is mounted on a folded flexible printed circuit board (F-PCB), embedded with UV and blue light-emitting diodes (LEDs) and a reference resistor for monitoring the gap state. Further details of the T-SPG and F-PCB designs are provided in fig. S7.

Having established the tunability of the *P*_*th*_ in the T-SPG device, we next demonstrate its ability to learn from a prelabeled dataset for a binary classification task, where a single T-SPG device optimizes the *P*_*th*_ for sorting inputs into two categories. To perform this classification, the T-SPG device compares the input power corresponding to *U*_*gate*_ with its internal *P*_*th*_. The resulting outcome of this comparison (*Y*_*out*_) follows the equation

$$Y_{\text{out}}=\left\{ \begin{array}{c} 1,f\left(U_{\text{gate}}\right)\geq P_{\text{th}}\;\left(\mathrm{gap}\;\text{close}\right)\\ 0,f\left(U_{\text{gate}}\right)<P_{\text{th}}\;\left(\mathrm{gap}\;\text{open}\right) \end{array}\right.$$ (1)

**Fig. 2.**
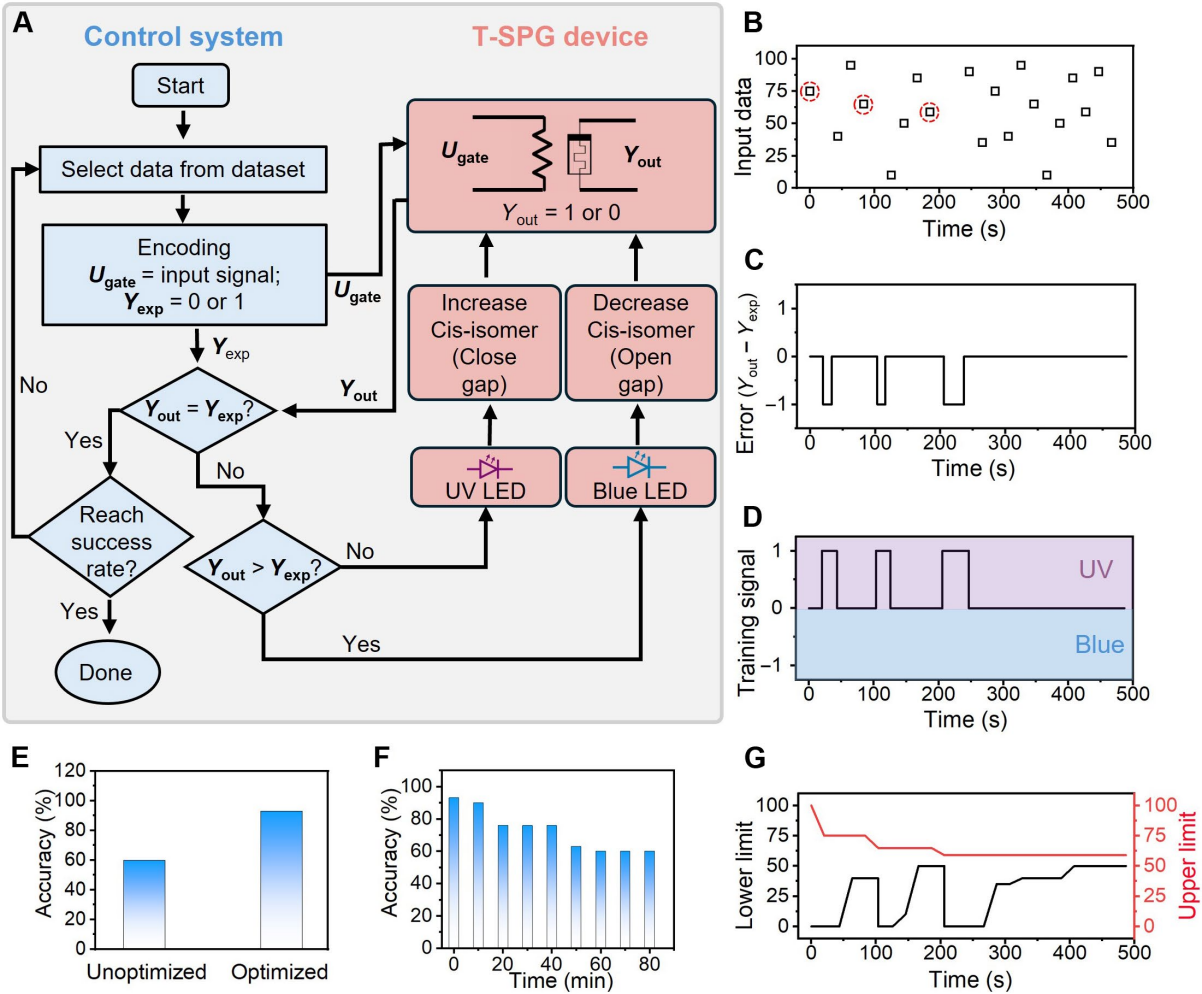
Training a single T-SPG device for binary classification task by optimizing its threshold power *P_th_*. (**A**) Operation flow diagram of the custom-built control system for optimizing the *P*_*th*_. (**B**) Sequence of the input of student grades over time; the red circles indicate data points that triggered optical correction. (**C**) Output error (*Y*_*out*_ − *Y*_*exp*_) for each data point during optimizing. (**D**) Corresponding optical modulation signal used to update the *P*_*th*_. (**E**) Comparison of classification accuracy before and after optimization. (**F**) Classification accuracy retention over time. (**G**) Evolution of the inferred passing score bounds over training; the upper (red) and lower (black) limit progressively converge to define the decision boundary.

If *Y*_*out*_ does not match the expected outcome (*Y*_*exp*_), the T-SPG needs to optimize *P*_*th*_ to enable *Y*_*out*_ = *Y*_*exp*_, through a custom-built external digital control system. As shown in Fig. 2A, this optimization process includes five steps: (i) encoding the prelabeled dataset into term of *U*_*gate*_ and their *Y*_*exp*_; (ii) applying this *U*_*gate*_ to the gating electrode of the T-SPG and monitoring the actual outcome *Y*_*out*_; (iii) comparing *Y*_*out*_ with *Y*_*exp*_; (iv) if a mismatch between *Y*_*out*_ and *Y*_*exp*_ is detected, activating UV or blue LEDs to tune *P*_*th*_ until *Y*_*out*_ = *Y*_*exp*_; (v) starting with next input from the dataset until the target success rate is reached. The circuit schematic and corresponding custom-built control system are provided in fig. S8.

As an illustrative example, we train the T-SPG device to infer the passing score from a prelabeled dataset of student exam grades (*G*). Initially, the passing score inferred by the T-SPG device is unknown, ranging from 0 to 100, which is progressively narrowed down through this optimization method. For optimization, the *G* is first encoded into a digital value (*x*), which defines a pulse-width modulated (PWM) *U*_*gate*_. This *x* value determines the average heating power delivered to the T-SPG. The encoding relation is defined as *x* = 1.62·*G* + 93, where 93 is an offset, which is an empirical constant compensating for the nonzero minimum of *P*_*th*_ under the low-intensity UV light (Fig. 1G). The 1.62 is a scaling factor, which maps the input score range (0 to 100) to the Arduino’s eight-bit PWM output range (0 to 255), taking the offset into account. The dataset and encoding formula are described in table S1. This PWM *U*_*gate*_ is applied to the T-SPG, inducing Joule heating to reduce gap width. The control system monitors the switch resistance in real time and assigns an output *Y*_*out*_: “1” (closed state) when the resistance falls below an empirically defined threshold of 1 kΩ, and “0” (open state) otherwise. This *Y*_*out*_ is then compared with the expected label *Y*_*exp*_, where 1 corresponds to a passing exam score and 0 to a failing one. If *Y*_*exp*_ = *Y*_*out*_, no light-modulation is required. If *Y*_*exp*_ >*Y*_*out*_, the UV LED is activated until the *Y*_*exp*_ = *Y*_*out*_. If *Y*_*exp*_ < *Y*_*out*_, the blue LED is activated.

To give a specific example, the first data point is *G* = 75, *Y*_*exp*_ = 1. The *G* is encoded into *x* = 214 by the equation given above. The corresponding PWM *U*_*gate*_ applied to the T-SPG has an average heating power of approximately 11.0 mW. This heating power raised the device temperature to around 35°C (fig. S9), which is insufficient to close the gap, causing *Y*_*out*_ = 0. The control system detects *Y*_*exp*_ >*Y*_*out*_, resulting in the activation of UV LED to close the gap (Fig. 2, C and D, and movie S3). An additional 10 s of irradiation is required to reach target stable state, which is caused by the photothermal effect due to the azobenzene, as described above and shown in Fig. 1D. Consequently, the upper boundary of the inferred passing score was updated downward from 100 to 75. The second data point is *G* = 40, *Y*_*exp*_ = 0, which is correctly classified, requiring no optical correction. As a result, the lower boundary of the inferred passing score was updated from 0 to 40. Following this, eight additional data points were sequentially tested. The optimization process was terminated once the success rate exceeded 90% for a test evaluating 10 data points. Over time, the inferred bounds progressively narrow, enabling more accurate estimation of the passing score (Fig. 2G). The final residual deviation is constrained by the dataset. Meanwhile, for a given consistent dataset, the convergence can only be progressed in one direction. In principle, the initial *P*_*th*_ determines the direction of optical modulation required: If initially set above the actual passing score boundary defined by the dataset, only UV irradiation is needed to lower *P*_*th*_; if set below, only blue irradiation is needed to raise *P*_*th*_ (fig. S10).

To quantify the optimization effectiveness, the trained T-SPG device is evaluated using a test dataset three times larger than the training set (table S2). Before optimization, the device achieved an initial classification accuracy of 60% (Fig. 2E). After optimization, the accuracy improved to 93%. This improvement, achieved through tuning the conversion to the cis-isomer of the azobenzene, highlights the effectiveness of the optimization process. Furthermore, it is notable that the Azo-LCON is also able to revert to its initial behavior over time, akin to a human being forgetting a memory (*52*, *53*). As shown in Fig. 2F, the T-SPG device slowly “forgets” the optimized *P*_*th*_, gradually reducing accuracy until it eventually returns to the initial state.

**Fig. 3.**
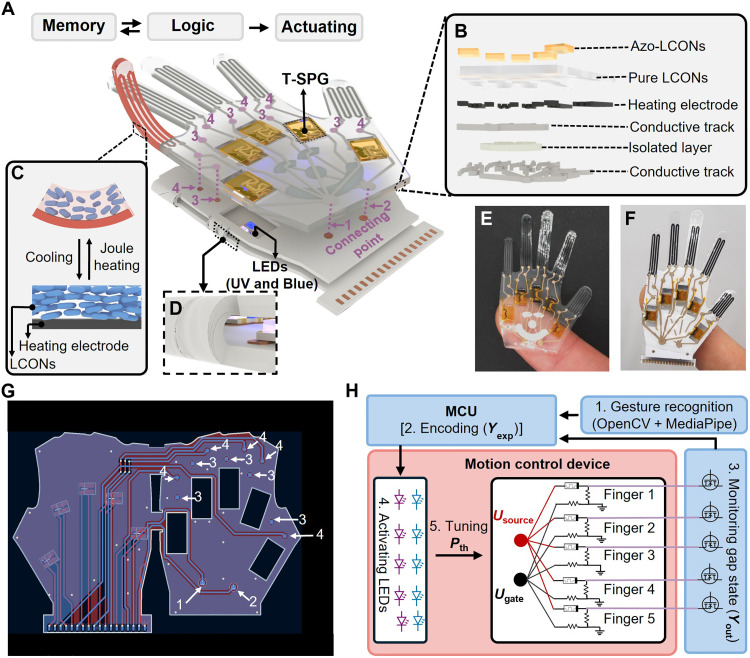
Design and functional overview of the motion control device. (**A**) Schematic of the motion control device, consisting of a printed artificial hand and a folded F-PCB embedded with 10 LEDs for optimizing the *P_th_* of five T-SPGs. Purple arrows indicate the electrical contact points between the artificial hand and the F-PCB, while markings 1 to 4 indicate applying *U_source_*, applying *U_gate_*, grounding, and monitoring the conductive switch state, respectively. (**B**) Exploded view of the printed artificial hand showing layer stack-up. (**C**) Schematic of the LCON-based actuator used as an artificial finger. (**D**) Zoomed-in schematic showing the folded F-PCB. (**E**) Printed artificial hand demonstrated on a human finger for the size comparison. (**F**) Final motion control device demonstrated on a human finger after assembling printed artificial hand and a folded F-PCB. (**G**) Circuit layout of the double-layer F-PCB before folding, where red and blue tracks represent the top and bottom electrode layers, respectively. Points 1 to 4 correspond to those in (A). (**H**) Schematic workflow for training the motion control device to reproduce human gestures, where the MCU represents the Arduino controller.

Having demonstrated the training of a single T-SPG for binary classification, we next integrate five T-SPGs with five LCON-based actuators into a motion control device in a hand-like configuration. As shown in Fig. 3A, this device comprises two main components: a printed artificial hand incorporating five T-SPGs and five actuators representing the bending finger. A folded F-PCB equipped with 10 LEDs is also included, with each T-SPG paired with one UV and one blue LED for *P*_*th*_ modulation. The LCON-based actuator consists of uniaxially aligned LCON with heating electrodes printed on the bottom (Fig. 3C). Each heating electrode is connected in series with its corresponding T-SPG gap, and all such series-connected pairs are wired in parallel to a common power source *U*_*source*_ via conductive tracks. This arrangement allows each T-SPG to switch its actuator on or off by controlling current flow. To enable localized Joule heating, the conductive ink, used for both the heating and gating electrodes, has a conductivity approximately 1000 times lower than that used for the conductive track and gap electrode. As a result, when the gap closes, current flows through the heating electrode, inducing LCON contraction. The heating electrode constrains this deformation, causing the film to bend toward the LCON side; the bending angle increases with increasing input power (fig. S11). Meanwhile, all T-SPG gating electrodes are connected in parallel to a common *U*_*gate*_ via conductive tracks. To prevent routing overlap for the *U*_*source*_ and *U*_*gate*_, an isolation layer is introduced between the two conductive tracks (Fig. 3B). The printed artificial hand is presented in Fig. 3E. More details about the design and fabrication are shown in fig. S12 and movie S4. The second component of the motion control device is a folded F-PCB with a double-layer circuit (Fig. 3D), where the red and blue tracks indicate the top and bottom circuit, respectively (Fig. 3G). The interface between the folded F-PCB and the printed artificial hand consists of four types of electrical contact points: for applying *U*_*source*_ for actuator power, applying *U*_*gate*_ for T-SPG, grounding, and monitoring the conductive switch state, which are labeled from 1 to 4 (Fig. 3, A and G). The final assembled motion control device is shown in Fig. 3F. More details about the F-PCB and assembling the motion control device are shown in fig. S13.

Next, we train the newly developed motion control device to reproduce human gestures through a conventional vision-based feedback control system that optimizes the *P*_*th*_ for each finger (Fig. 3H). The corresponding circuit diagram and the physical layout of the control system are provided in the fig. S8. We used Chinese number gestures ranging from “1” to “10” (*54*). A webcam continuously captured the user’s hand movements, and the gesture was recognized in real time by using OpenCV for image processing, MediaPipe for hand landmark detection and a custom-trained machine-learning model to convert landmarks to gestures (fig. S14). The captured gesture is sent to the Arduino microcontroller unit (MCU), which is encoded into a five-element vector *Y*_*exp*_. Each element of *Y*_*exp*_ corresponds to a finger, where a value of 1 denotes a bent finger and 0 indicates a straight one. Consequently, the MCU activates the corresponding LEDs to optimize the *P*_*th*_ if a mismatch is detected between the output vector *Y*_*out*_ and the expected vector *Y*_*exp*_.

**Fig. 4.**
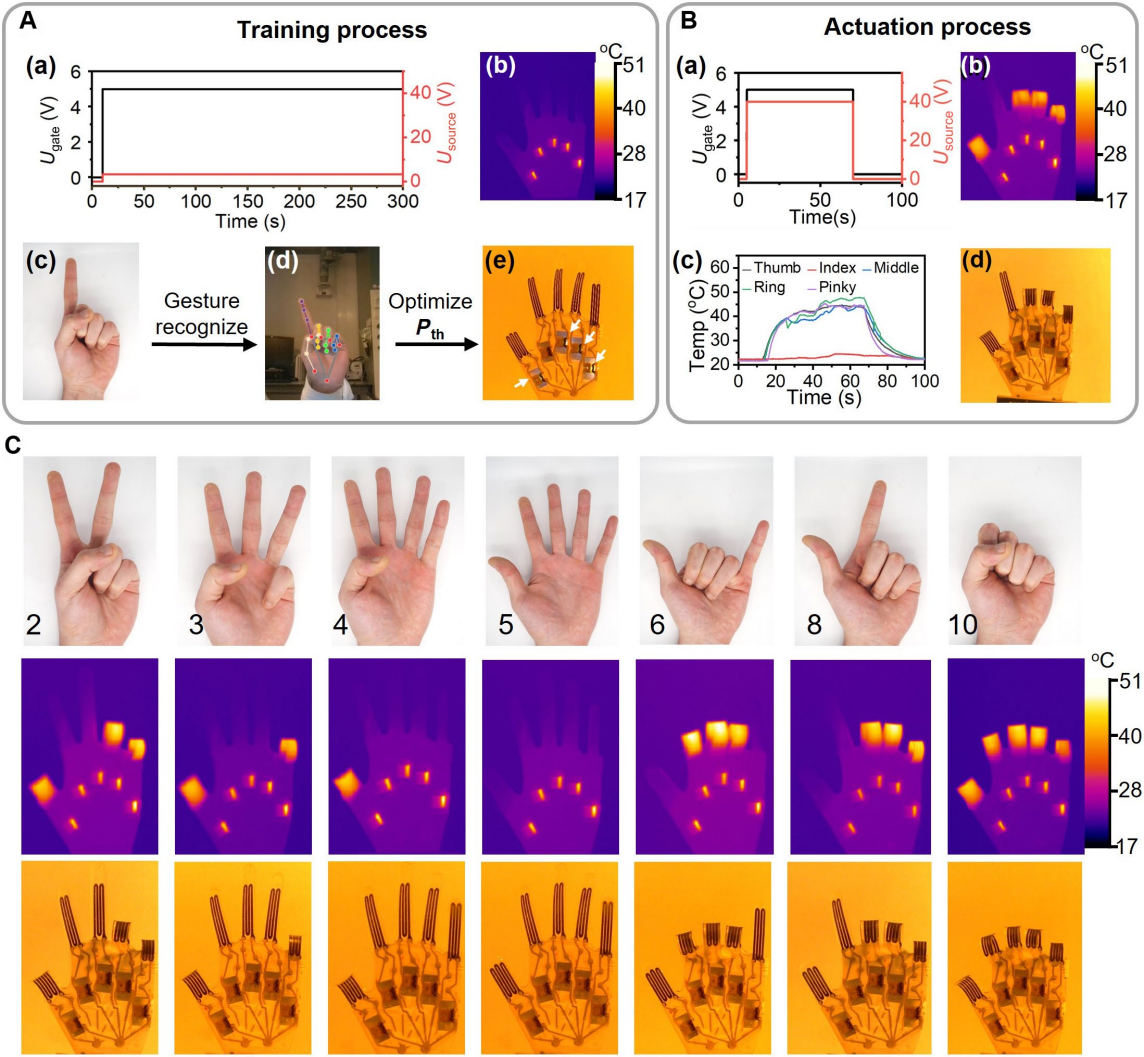
Training the motion control device to reproduce Chinese number gestures. (**A**) Training process of the motion control device. (a) The input signals *U*_*gate*_ and *U*_*source*_ applied during training. (b) Corresponding thermal images showing uniform localized heating at five T-SPGs. (c) Target Chinese number gesture “1” as input. (d) Real-time gesture recognition. (e) UV activation for the specific T-SPGs based on the recognized gestures; white arrows indicate positions of UV LEDs that are turned on. (**B**) Actuation process of the motion control device after training. (a) The input *U*_*gate*_ and *U*_*source*_ applied to the motion control device during actuation. (b) Thermal images showing Joule heating in specific fingers. (c) Temperature profiles of each finger (thumb to pinky) during actuation. (d) Photograph of the motion control device reproducing the Chinese number gesture 1. (**C**) Demonstration of the motion control device reproducing Chinese number gestures “2” to “6,” “8,” and “10.” Each column shows, in order, a photograph of the target gesture, a thermal image of the reproduced gesture, and a photograph of the reproduced gesture.

As a representative case, we use the Chinese number gesture 1 (Fig. 4, A and B) for training. To reduce the optimization steps of the *P*_*th*_, a constant 5.0-V direct voltage is applied as the *U*_*gate*_, corresponding to an input power of approximately 10 mW per T-SPG unit. Meanwhile, a constant 3.3 V is applied as *U*_*source*_ to enable real-time monitoring of the switch states [Fig. 4A (b)]. Before optimizing the *P*_*th*_, the motion control device was allowed to stabilize for approximately 20 s, during which all five T-SPGs reached a uniform and identifiable temperature profile [Fig. 4A (b) and fig. S15]. After stabilization, the MCU activates the UV LEDs to decrease the *P*_*th*_ of the T-SPGs associated with the thumb, middle, ring, and pinky fingers [Fig. 4A, (c) to (e)] until their *Y*_*out*_ = *Y*_*exp*_. More details about the training process are shown in fig. S16 and movie S5. After training, the motion control device is detached from the control system. The motion control device can now present gesture 1 under the same *U*_*gate*_ and *U*_*source*_. Because the motion control device can be detached from the control system, a higher *U*_*source*_ can be applied, leading to a more pronounced deformation (Fig. 4B); these trained gestures can be repeated more than 10 times. To further demonstrate the functional range of our motion control device, Chinese number gestures 2 to 6, 8, and 10 are successfully trained and reproduced using the same input conditions as shown in Fig. 4C and movie S6. Because of the sensitivity of the azobenzene to ambient light, all photographs of the motion control device were taken under yellow light to avoid unintended UV exposure.

## DISCUSSION

We have developed an intelligent device based on a data-trainable LCON that integrates logic, memory, and actuation within a single material system. To achieve this, the LCON was functionalized with azobenzene and engineered into a T-SPG, a component that uniquely has both logic and memory functionalities. The T-SPG is trained by a conventional computing system, allowing a learned state to be stored directly in the LCON’s molecular configuration. The significance of our T-SPG design is that the processed output can directly command an actuator without requiring external signal amplification. This capability enabled us to create a motion control device by directly integrating five T-SPGs with five LCON actuators, demonstrating the ability to reproduce Chinese number gestures after training. The current motion control device cannot, yet, directly reproduce gestures “7” and “9” under the same input conditions. Gesture 7 can only be realized by lowering the *U*_*source*_ during actuating, while gesture 9 would require an independent *U*_*source*_ for each individual finger to trigger different actuation magnitudes. These limitations stem from the binary nature of the current T-SPG design, which provides only on/off control for each finger. Extending this concept to multivalued or analog logic, where each finger could exhibit graded bending, would enable more complex gestures and represents an important direction for future research. The functional capacity of our LCON systems will be expanded in our vision for our device. Nonetheless, the T-SPG provides the precedent for material intelligence. It often operates through abstract physical states, such as mechanical deformation or molecular orientation, which inspires new design possibilities for self-learning materials.

## MATERIALS AND METHODS

### Materials

The 2-methyl-1,4-phenylene bis(4-(3-(acryloyloxy)propoxy)benzoate) was purchased from Merck. The 4-(6-(acryloyloxy)hexyloxy)phenyl 4-(6-(acryloyloxy)hexyloxy)benzoate was obtained from Daken Chemical Limited. The 4,4′-bis(6-acryloyloxyhexyloxy)azobenzene was supplied by Synthon Chemicals. The 2,2′-(ethylenedioxy)diethanethiol, butylated hydroxytoluene, and 1,8-diazabicyclo[5.4.0]undec-7-ene (DBU) were purchased from Sigma-Aldrich. Bis[2,6-difluoro-3-(1H-pyrrol-1-yl)phenyl]titanocene (Irgacure 819) was obtained from abcr GmbH (Germany). Polyvinyl alcohol (PVA, average molecular weight ≈25,000 g/mol) was purchased from Sigma-Aldrich. Dichloromethane (DCM) was supplied by Biosolve. Two commercial conductive inks (SH5025 and SC1502) and a stretchable insulating ink (SI3104) were obtained from ACI Materials Inc. An additional conductive ink (Elepaste NP1) was acquired from Taiyo. All materials were used as received without further purification.

### Synthesis of azobenzene-functionalized liquid crystal oligomer

Azobenzene-functionalized oligomer was synthesized via a base-catalyzed thiol-acrylate Michael addition reaction. The chemical structures of the five molecules used in the azobenzene-containing formulation are shown in fig. S1A. The total molar ratio of acrylate to thiol groups was approximately 1:0.85, with 10 mol % of the acrylate functionality contributed by a diacrylate-functionalized azobenzene derivative. First, molecule 3 (209 mg) and molecule 4 (619 mg) were combined in a glass vial and dissolved in 5 ml of DCM. The catalyst DBU (5 μl) was added, and the mixture was stirred for 30 min at room temperature. In parallel, molecule 1 (1412 mg) and molecule 2 (646 mg) were dissolved in another 5 ml of DCM in a separate vial. This solution was then added to the first vial, followed by an additional 5 μl of DBU. The reaction was allowed to proceed overnight under ambient conditions. Subsequently, molecule 5 (90 mg) was added to the reaction mixture, and the vial was covered with aluminium foil to protect it from light. The mixture was stirred until complete dissolution of molecule 5. The resulting solution was then transferred to a polytetrafluoroethylene (PTFE) evaporation dish and placed in a vacuum oven at 45°C overnight to remove most of the DCM solvent. The dried oligomer film was then manually flipped and returned to the vacuum oven for an additional 3 hours to eliminate residual solvent. The final oligomer product was collected and loaded into a syringe for subsequent four-dimensional (4D) extrusion printing.

### Synthesis of pure liquid crystal oligomer

The pure liquid crystal oligomers were synthesized via a base-catalyzed thiol-acrylate Michael addition reaction as well. The chemical structures of the molecules used in the pure-LCON formulation are shown in fig. S17. The total molar ratio of acrylate to thiol groups was approximately 1:0.8. First, molecule 1 (1412.6 mg), molecule 2 (861.7 mg), molecule 3 (663.6 mg), molecule 4 (2.5 mg), and molecule 5 (20 mg) were combined in a glass vial. Next, 10 ml of DCM was added to dissolve the mixture, followed by 10 μl of DBU as the base catalyst. The solution was stirred for 3 hours at room temperature and then left overnight to complete the reaction. The resulting mixture was transferred to a PTFE evaporation dish and placed in a vacuum oven at 45°C overnight to remove most of the DCM. The dried oligomer film was then manually flipped and returned to the vacuum oven for an additional 3 hours to eliminate residual solvent. The final oligomer was collected and loaded into an ink syringe for subsequent 4D extrusion printing.

### Preparation of the conductive inks

Conductive ink for printing gating electrodes was prepared by blending the SH5025 and SC1502. The two conductive inks were directly added to the vial and mechanically stirred for 10 min. Typically, a mixture containing 40 wt % SH5025 and 60 wt % SC1502 was used for preparing the gating electrode of the T-SPG and the heating electrode of the LCON actuator. For printing the gap electrode, a pure commercial conductive ink (Elepaste NP1) was used. Last, the two tailored conductive inks were loaded into separate syringes.

### The fabrication of the T-SPG device

The T-SPG devices were fabricated using a multistep direct ink writing (DIW) process. The layer-by-layer fabrication began by printing the conductive gating and gap electrodes onto a sacrificial PVA layer. Subsequently, the active azobenzene-functionalized LCON (Azo-LCON) and the surrounding pure LCON matrix were printed on top of the electrodes. Last, the printed film was peeled from the substrate after dissolving the sacrificial layer and then assembled with an F-PCB. Full fabrication details are provided in fig. S7.

### The fabrication of the motion control device

The fabrication of the motion control device followed a multistep DIW procedure similar to that of the single T-SPG devices but with increased complexity to create the five-finger hand structure. The process involved the sequential printing of several functional layers onto a sacrificial substrate. First, a two-layer circuit architecture was constructed by printing multiple layers of conductive and stretchable insulating inks to define the gating, heating, and gap electrodes for all five T-SPG units and finger actuators. Subsequently, two different liquid crystal oligomers were precisely deposited: The Azo-LCON was printed in the T-SPG regions, while pure LCON was used to form the finger actuators and hand body. After curing, the monolithic device was detached from the substrate. A detailed, step-by-step protocol is provided in fig. S12. After printing the artificial LCON hand and cutting the micro gap in the T-SPGs, the LCON sample is manually assembled with a folded F-PCB integrated with one UV LED and one blue LED per T-SPG unit. The circuit layout and assembly procedure are detailed in fig. S13.

### Optical characterization

The alignment of the printed LCON films and the microgap structures was examined using a polarized optical microscope (Leica DM2700M). The light intensity of UV and blue LED is measured by radiometer (Opsytec Dr. Groebel GmbH). The gap width changes under varying electrical and optical inputs were quantitatively analyzed using a custom Python script based on the OpenCV library. The UV-Vis absorption spectra of Azo-LCON were measured using a PerkinElmer Lambda 750 UV/Vis/NIR spectrophotometer. Photographs and movies were acquired using a digital camera (Olympus E-M10 Mark IV with a 60-mm lens) and a USB camera (2 MP, 60 fps). Thermal images and movies were captured using an infrared thermal imaging camera (Xenics Gobi+ 640 GigE).

### Dynamic mechanical thermal analysis

Dynamic mechanical thermal analysis was performed using a TA Instruments Q800 device in vertical tension mode to characterize the thermomechanical response of uniaxially aligned Azo-LCON thin films. The measurements were conducted in force-controlled mode to evaluate thermal strain behavior. Samples were first cooled to 0°C and held for 5 min under a preload force of 0.005 N. The same static force was maintained while the samples were heated to 70°C and subsequently cooled back to 0°C at a rate of 5°C/min. This heating-cooling cycle was repeated twice to eliminate thermal history effects.

### WAXS analysis

Wide-angle x-ray scattering (WAXS) measurements were carried out using a Ganesha Lab instrument equipped with a Genix-Cu ultralow divergence x-ray source (λ = 0.154 nm, flux = 1 × 10^8^ photons s^−1^). Diffraction patterns were recorded using a Pilatus 300 K silicon pixel detector (487 pixels by 619 pixels, pixel size: 172 μm by 172 μm). Silver behenate was used as a calibration standard. The sample-to-detector distance was set to 89 mm, enabling the determination of the order parameter of LCON. The instrument was equipped with in situ UV and blue LEDs, allowing real-time observation of order parameter changes under light stimulation. The intensity of the UV and blue light was 3.1 and 1.8 mW cm^−2^, respectively.

### Electrical characterization

The source-drain current of the T-SPG was measured under varying input power conditions using a source meter (Keithley, 2400). A dc power supply (TENMA, 72-2720) provided the gating voltage, while MATLAB (R2019a) served as the control platform for synchronized signal delivery and data acquisition. A multichannel programmable power source (Moku:Go, Liquid Instruments) was used to supply the UV and blue LEDs during optical modulation measurements of *P*_*th*_ in the T-SPG. For the training and actuation of the motion control device, the same DC power supply was used to provide *U*_*source*_, while *U*_*gate*_ was delivered through the Moku:Go platform.

### Training the single T-SPG for binary classification

The entire training process, including PWM signal generation, real-time resistance monitoring, output classification, and UV/blue LED activation, was executed autonomously via an Arduino microcontroller (Arduino, NANO 33 BLE). The circuit design of the training system can be found in fig. S8. The full code can be found on Zenodo (*55*).

### Gesture recognition and training procedure

Gesture recognition was performed using a vision-based system that combined OpenCV and MediaPipe in Python to detect hand landmarks and classify hand gestures in real time. The recognized gesture was transmitted as a string identifier (e.g., “FIVE”) to an Arduino controller via serial communication. Upon receiving the identified gesture, the Arduino encoded it into an expected binary output vector *Y*_*exp*_ corresponding to the target finger activation pattern. The Arduino then compared *Y*_*exp*_ to the actual device output *Y*_*out*_ based on resistance values of the five T-SPGs. If a mismatch was detected, the system automatically activated the UV or blue LEDs associated with the corresponding fingers to adjust the *P*_*th*_. This closed-loop training procedure was fully automated. The circuit design of the vision-based training system is shown in fig. S8. The full Python and Arduino code is available on Zenodo (*55*).
