# Supplementary material for: Trainable soft electronics with memory in liquid crystal polymers
Source: Sci Adv. 2026 Jul 8;12(28):eaee8616. doi: 10.1126/sciadv.aee8616 (PMC13344293; doi:10.1126/sciadv.aee8616)
Supplement: Supplementary file 1 — Tables S1 and S2 Figs. S1 to S17 Legends for movies S1 to S6 [file sciadv.aee8616_sm.pdf]

Science Advances
AAAS

Supplementary Materials for

Trainable soft electronics with memory in liquid crystal polymers

Pengrong Lyu *et al.*

Corresponding author: Danqing Liu, danqing.liu@tue.nl

Sci. Adv. **12**, eaee8616 (2026)
DOI: 10.1126/sciadv.aee8616

The PDF file includes:

Tables S1 and S2
Figs. S1 to S17
Legends for movies S1 to S6

Other Supplementary Material for this manuscript includes the following:

Movies S1 to S6

Supplementary Tables

Table S1: Training dataset and encoding formula for binary classification task.

For the binary classification task presented in Fig. 2, we used a dataset of 10 student exam grades (**G**, ranging from 0 to 100) to train the T-SPG device, as detailed in Table 1. Each grade was pre-labelled with an expected classification output (Y_{exp}), where '1' denotes a passing score and '0' denotes a failing score. The classification threshold for passing the exam in the dataset was set at 55. To input the numerical grade (G) to the T-SPG, it needed to be encoded into an electrical signal that the T-SPG could process, specifically input electrical power. To simplify the Arduino-based control system, we fixed the input voltage at V_{in} and utilized a pulse-width modulated (PWM) signal (U_{gate}) to control the input power to the T-SPG. The PWM signal generated by the Arduino microcontroller has an 8-bit resolution, corresponding to a value x in the range of 0 to 255. Thus, the input power (P_{in}) is given by $P_{in} = x/255 * V_{in}^2/R_1$, where R_1 is the resistance of the heating electrode.

Next, we used a basic linear function to encode G into x, defining a linear encoding relation of the form: $x = A \cdot G + b$, where A is a scaling factor and b is an offset. According to the experimental results presented in Fig. 1G, the effective reversible tuning range of the threshold power (P_{th}) through alternating UV and blue light irradiation was determined to be 8.55 mW – 12.00 mW. We set the maximum P_{th} to correspond to the maximum x. Thus, the tuneable range for x was determined to be 181 – 255, which was set to map the grade range for passing the exam (55–100). By solving for A and b using these boundary conditions, we obtained A = 1.62 and b = 93. This encoding ensures that U_{gate} can accurately regulates the average heating power delivered to the T-SPG. It should be noted that if the tuneable range of P_{th} changes, the encoding equation must be adjusted correspondingly.

**Table S1**										
**Student**	**1**	**2**	**3**	**4**	**5**	**6**	**7**	**8**	**9**	**10**
**Grade**	75	40	95	65	10	50	85	59	90	35
Y_{exp} (Pass =1 or Fail=0)	1	0	1	1	0	0	1	1	1	0

Table S2: The dataset used for testing the classification accuracy

To quantify classification accuracy, the trained T-SPG device was evaluated using a test dataset three times larger than the training set. This data was randomly generated as integers ranging from 0 to 100. The corresponding dataset is presented in Table S2.

Table S2										
**Student**	**1**	**2**	**3**	**4**	**5**	**6**	**7**	**8**	**9**	**10**
**Grade**	33	43	85	29	0	8	85	56	19	11
Y_{exp}	0	0	1	0	0	0	1	1	1	0
**Student**	**11**	**12**	**13**	**14**	**15**	**16**	**17**	**18**	**19**	**20**
**Grade**	17	32	79	66	30	93	56	5	43	51
Y_{exp}	0	0	1	1	0	1	1	0	0	0
**Student**	**21**	**22**	**23**	**24**	**25**	**26**	**27**	**28**	**29**	**30**
**Grade**	69	53	64	92	49	23	80	68	72	47
Y_{exp}	1	0	1	1	0	0	1	1	1	0

Supplementary Figures

Fig. S1. Chemical composition, alignment, and thermomechanical properties of the Azo-LCON.

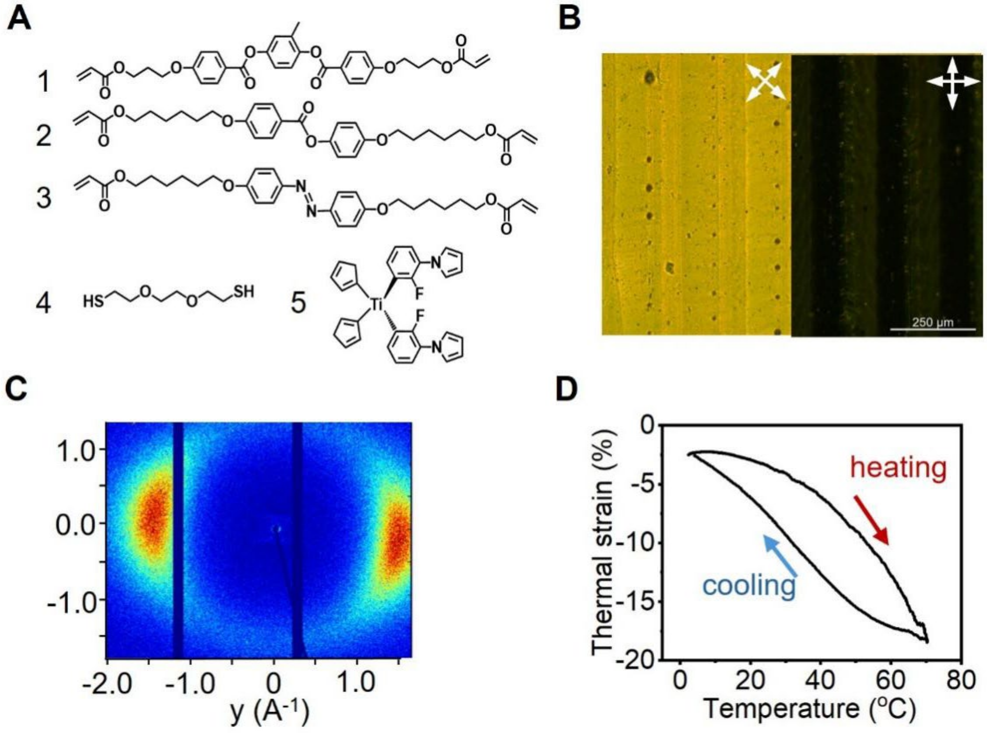

Fig. S1. Chemical composition, alignment, and thermomechanical properties of the Azo-LCON. A, Chemical structures of the molecules used to synthesize the azobenzene-functionalized liquid crystal oligomer networks (Azo-LCON). **B**, Polarized optical microscopy (POM) image of a printed Azo-LCON film, showing uniform birefringence that indicates good uniaxial alignment. Scale bar, 250 µm. **C,** Two-dimensional Wide-Angle X-ray Diffraction (2D-WAXD) pattern of an Azo-LCON film at room temperature, confirming the uniaxial alignment of the liquid crystal mesogens, with a calculated scalar order parameter (S) of 0.43. **D**, Thermal strain of an Azo-LCON film, measured parallel to the director, as a function of temperature. The red and blue curves indicate the heating and cooling cycles, respectively, showing a large and reversible actuation strain.

Fig. S2. Influence of fabrication conditions on the initial gap width of the T-SPG.

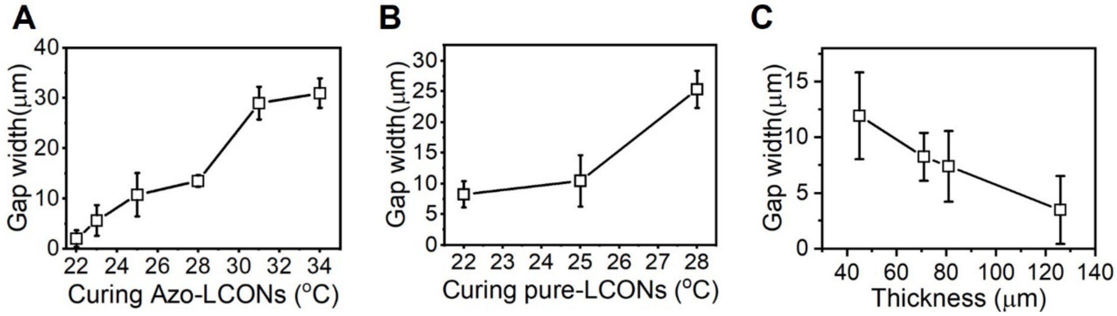

Fig. S2. Influence of fabrication conditions on the initial gap width of the T-SPG. A, Gap width as a function of the curing temperature for Azo-LCON. (Fixed parameters: Pure LCON cured at ~25°C; Azo-LCON thickness ~80 μm). **B**, Gap width as a function of the curing temperature for pure-LCON. (Fixed parameters: Azo-LCON cured at ~28°C; Azo-LCON thickness ~80 μm). **C**, Gap width as a function of the thickness for Azo-LCON. (Fixed parameters: Azo-LCON cured at ~28°C; pure LCON cured at ~25°C).

Fig. S3. Optical properties and alignment change of Azo-LCON under light irradiation.

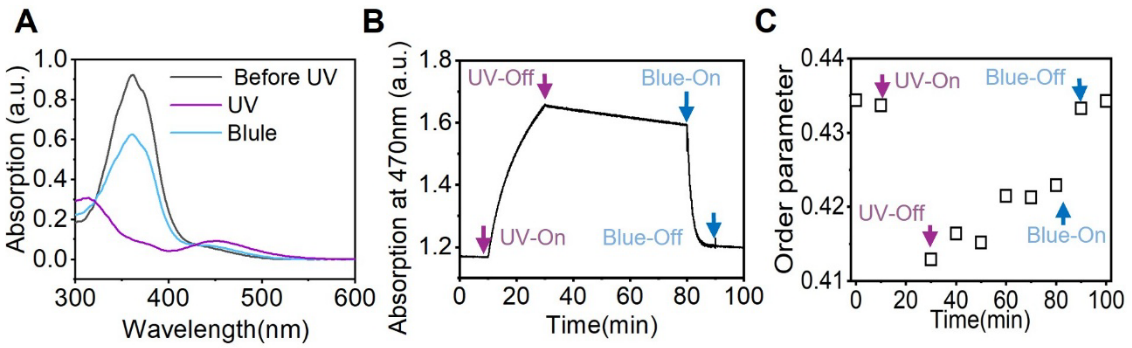

Fig. S3. Optical properties and alignment change of Azo-LCON under light irradiation. A, Normalized absorption spectra of a uniaxially aligned Azo-LCON film (containing 10 mol% azobenzene molecules) before UV irradiation (black), after UV irradiation (365 nm, 20 mW/cm², 10 min; magenta), and after blue irradiation (455 nm, 15 mW/cm², 10 min; cyan). **B**, Time-dependent absorption (at 470 nm) of the uniaxially aligned Azo-LCON. Purple arrows (UV-On/Off) and blue arrows (Blue-On/Off) indicate the switching on and off of UV light (365 nm, 3.0 mW/cm²) and blue light (455 nm, 1.8 mW/cm²), respectively. **C**, Corresponding change in the scalar order parameter of the uniaxially aligned Azo-LCON. Purple arrows (UV-On/Off) and blue arrows (Blue-On/Off) indicate the switching on and off of UV light (365 nm, 3.0 mW/cm²) and blue light (455 nm, 1.8 mW/cm²), respectively.

Fig. S4. Thermal relaxation of azobenzene molecules in Azo-LCON and gap width relaxation under dark conditions.

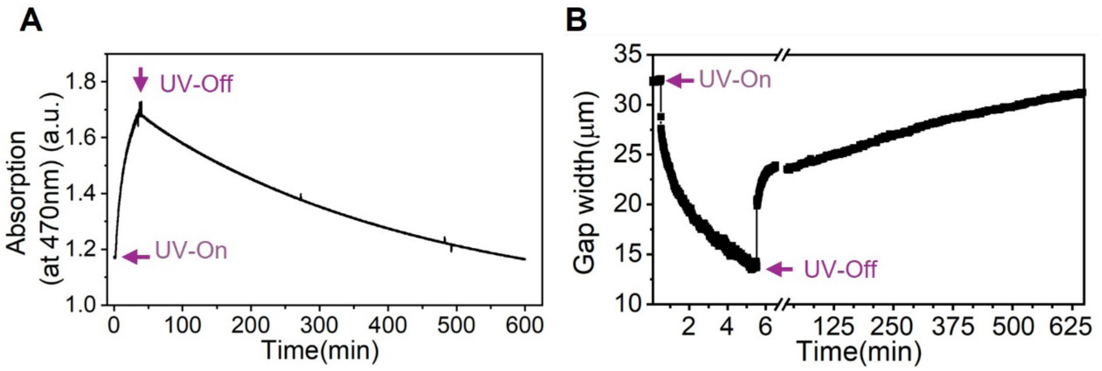

Fig. S4. Thermal relaxation of azobenzene molecules in Azo-LCON and gap width relaxation under dark conditions. **A**, Time-dependent absorption (at 470 nm) of the uniaxially aligned Azo-LCON in a dark environment. The purple arrows indicate the times when the UV light was switched on or off. **B**, Time-dependent gap width of the T-SPG under dark conditions.

Fig. S5. Influence of blue light intensity on the T-SPG's actuation dynamics and photothermal response.

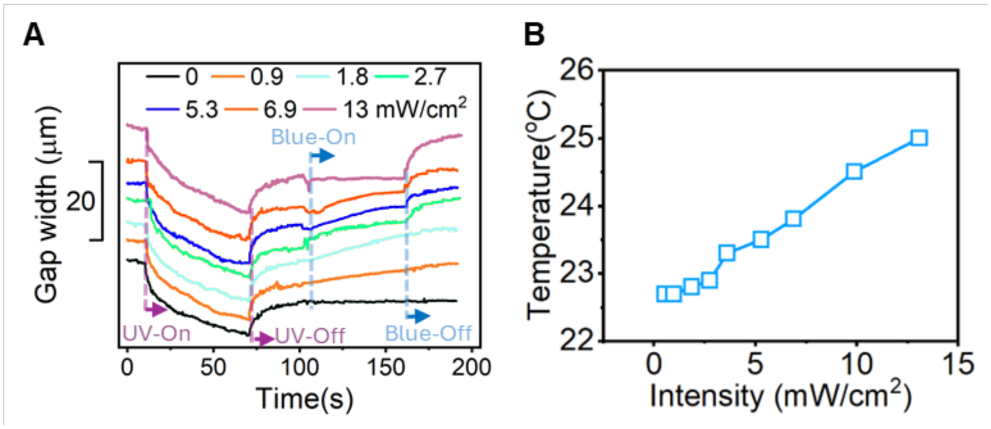

Fig. S5. Influence of blue light intensity on the T-SPG's actuation dynamics and photothermal response. A, Gap width of the T-SPG as a function of time under different blue light irradiation intensities. All samples were first irradiated with a fixed UV light intensity (365 nm, 15.6 mW/cm²) to close the gap. The result shows that while low-intensity blue light (e.g., 1.8 mW/cm²) effectively reopens the gap, higher intensities induce a competing photothermal effect that counteracts the reopening, leading to incomplete recovery. Purple arrows indicate the switching on and off of UV light (365 nm, fixed intensity of 15.6 mW/cm²). Blue arrows indicate the switching on and off of blue light (455 nm) at various intensities, as specified in the legend. **B**, Measured temperature of the Azo-LCON film as a function of the irradiating blue light intensity.

Fig. S6. The reliability and experimental reversibility of T-SPG device.

The training behaviour of the T-SPG is based on the isomerization of azobenzene, and thus the reversibility and lifetime of the system are highly temperature-dependent. We discuss the reliability of the T-SPG from two aspects:

1. **Reversibility under UV/blue-light training (write-erase cycles) at room temperature:** To assess the cyclic stability of the T-SPG under optical training, we performed repeated write-erase cycles in the dark (room temperature 20ºC), as shown in Fig. S6. A typical cycle consisted of UV irradiation (3.0 mW/cm², 20 min) to close the gap, followed by a short electrical measurement (within 1 min) to determine the Pth, and then blue light irradiation (1.8 mW/cm², 10 min) to reopen the gap, again followed by a 1 min electrical measurement. We observed that the $\boldsymbol{P_{th}}$ values remained stable for over 50 cycles. This indicates that under the standard training protocol (where each cycle involves only short Joule heating for characterization), the azo-LCON exhibits good reversibility.
2. **Lifetime and memory retention under repeated electrical activation (under joule heating):** The lifetime of the system is highly temperature-dependent. Repeated heating accelerates the cis-to-trans isomerization of azobenzene, leading to gradual forgetting of the trained $\boldsymbol{P_{th}}$. This behavior was evaluated in main text Fig. 2G of our manuscript. After training the device with UV light, we evaluated its classification accuracy (linked to the gap size and $\boldsymbol{P_{th}}$) using a test dataset consisting of 30 data points, each requiring a 20s electrical heating pulse. The accuracy gradually decreased over successive rounds of testing. After 7 rounds (i.e., 7 × 30 = 210 heating pulses), the accuracy returned to its initial untrained level, indicating that the gap size and $\boldsymbol{P_{th}}$ had fully recovered to its original value. Thus, our data show that in the dark, the trained memory can withstand approximately 210 heating cycles before being completely erased. This thermal sensitivity represents a limitation of the current system that would need to be addressed for applications requiring long-term memory retention or operation under varying thermal conditions.

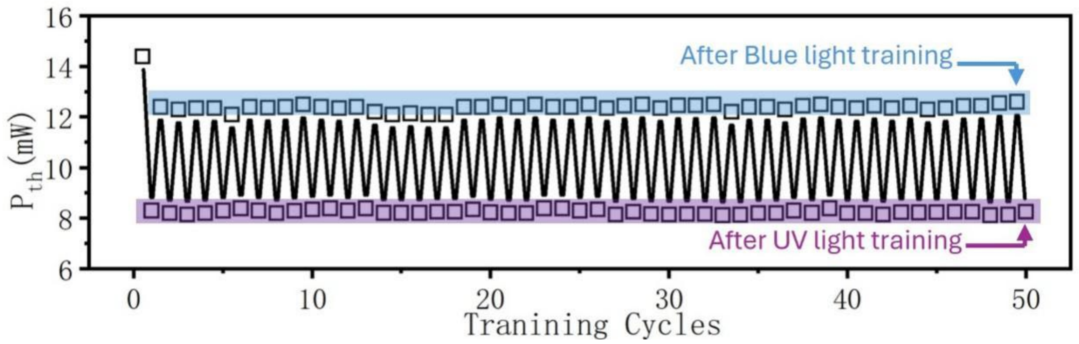

Fig. S6. Reversibility of the P_{th} under repeated optical training.

Fig. S7. Detailed design and assembly of the T-SPG device.

The T-SPG devices were fabricated using a multi-step direct ink writing (DIW) process. The print paths are illustrated in Fig. S7. The process began with the preparation of a sacrificial layer by spin-coating (2000 rpm for 30s) a 10 wt% polyvinyl alcohol (PVA) solution onto a pre-cleaned microscope slide. The subsequent printing steps were performed layer-by-layer:

1. **Gating Electrode:** A conductive ink blend was printed and cured at 120 °C for 30 minutes.
2. **Gap Electrode:** A second conductive ink (Elepaste NP1) was printed and thermally cured at 90 °C for 20 minutes. Both electrode layers were printed using a 150 μm nozzle at a speed of 250 mm/min.
3. **Azo-LCON Layer:** The azobenzene-functionalized LCON was printed over the electrodes using a 300 μm nozzle at 400 mm/min, with the print head at 40 °C and the substrate at 25 °C. This layer was then photo-crosslinked under a nitrogen atmosphere using a 565 nm green LED for 1 hour. The standard curing temperature was typically 28 °C. For experiments investigating the influence of fabrication conditions, this temperature was systematically varied.
4. **Surrounding Matrix:** Pure-LCON was printed around the active region and subsequently photo-crosslinked for 20 minutes under a nitrogen atmosphere using a filtered UV light source ($\lambda > 400$ nm). The curing temperature is typically set at 25 ºC.

After printing and curing, the samples were immersed in water for 3 hours to dissolve the sacrificial PVA layer, and carefully peeled off from the substrate. The detached films were air-dried to remove residual water. Then, a handmade die was used to cut through both the gap electrodes and the LCON film, thereby forming the initial micro-gap. This step was performed at an elevated temperature (typically 45 °C), allowing the gap to further open upon cooling to room temperature. Finally, the printed T-SPG films were manually assembled with a folded flexible printed circuit board (F-PCB) integrated with one UV LED, one blue LED and one reference resistor.

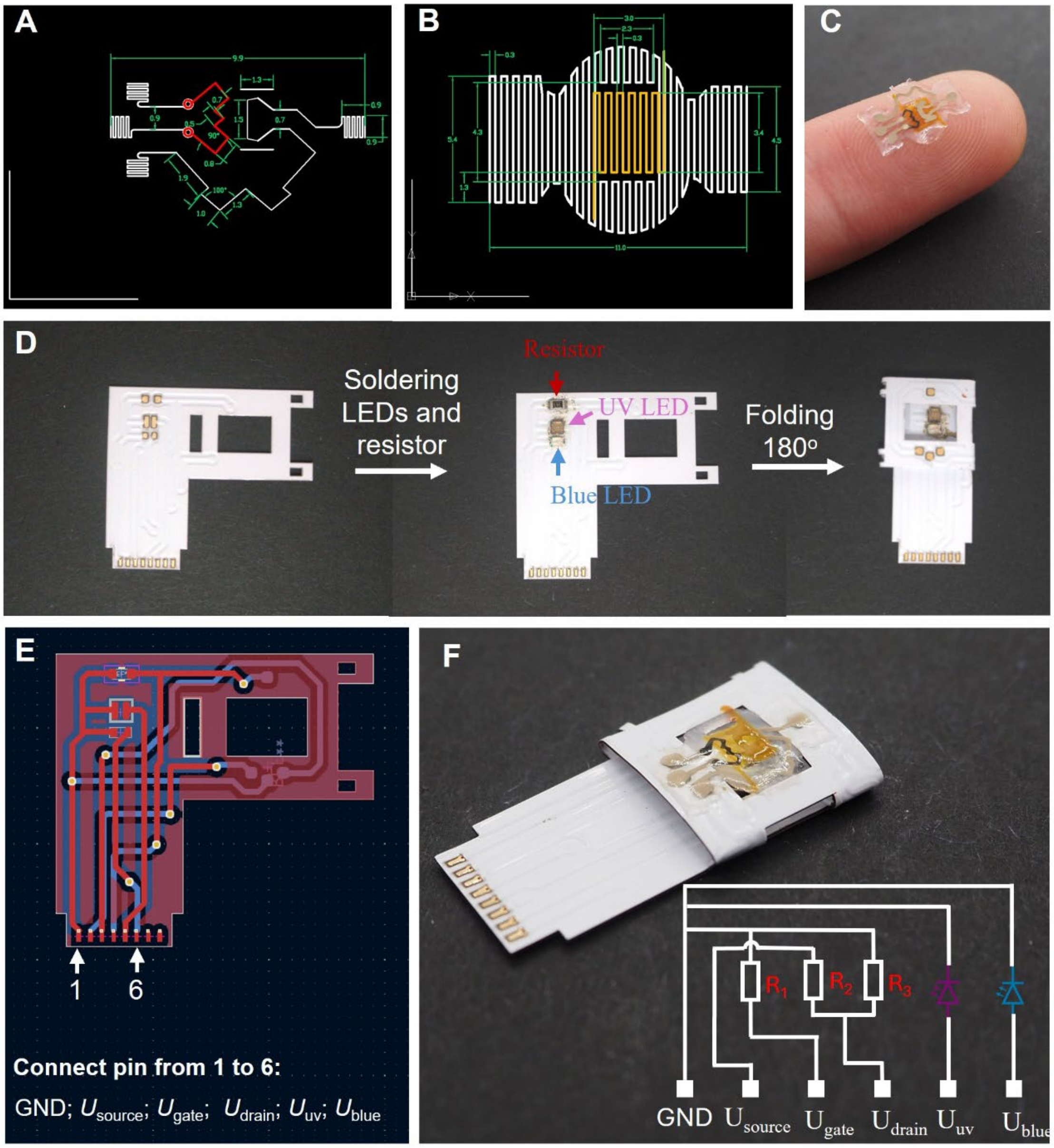

Fig. S7. Detailed design and assembly of the T-SPG device. **A,** The dimensions and printing paths for the gating electrode (red), and gap electrode (white). **B**, The dimensions and printing paths for the Azo-LCON (yellow) and surrounding pure LCON (white) layers. **C**, Photograph of the flexible, printed T-SPG film on a human finger. **D**, Assembly sequence of the F-PCB, showing the board before soldering, after mounting LEDs and a resistor (39 kΩ), and after folding. **E**, Circuit layout of the double-layer F-PCB, with red and blue tracks representing the top and bottom layers, respectively. There are six key electrical terminals: **GND** (grounding), $\boldsymbol{U_{source}}$, $\boldsymbol{U_{drain}}$ (output signal for gap

monitoring), $\boldsymbol{U_{gate}}$ (input signal for gap modulation), $\boldsymbol{U_{\mathbf{uv}}}$ (input signal for UV LED), $\boldsymbol{U_{blue}}$ (input signal for blue LED). **F**, The final assembled T-SPG device after integrating the printed film with the folded F-PCB. Inset**:** The equivalent circuit diagram of the T-SPG device. R_1, R_2, and R_3 correspond to the resistance in the gating electrode, gap electrode, and reference resistor(39 kΩ), respectively.

Fig. S8. Custom-built digital control system for training the T-SPG device and motion control device.

Our custom-built digital control system, based on an Arduino microcontroller, is designed to support two distinct applications. The first is the training and testing of a single T-SPG device for binary classification task, and the second is the training of the motion control device for gesture replication task. Although the core hardware is shared, its operational logic and power management are tailored for each application as detailed below.

- **Mode 1: Training and testing the single T-SPG for binary classification task**

  The Arduino microcontroller converts pre-labelled into a combination of Pulse-Width-Modulation (PWM) signal for the gating electrode (GapHeater) and expected reading on the sensor pin ($\boldsymbol{U}_{\mathrm{drain}}$ from the T-SPG device). The Arduino's LED signal pins provide 3.3V 3mA each, which is sufficient to directly power the LEDs without amplification. PWM signal can be used on the LED pins to tune the LED intensity as well. The GapHeater and ActuatorHeater pins provide PWM signals that are amplified by gate drivers and subsequently fed into the gates of high-side N-type MOSFETs. 12V is supplied to the gate drivers by a small 5V-12V boost converter. Power for the GapHeater is provided by an external power supply. For the T-SPG, power for the ActuatorHeater is provided by the Arduino's 3.3V line. There is no actuator connected after the gap, only a pull-down resistor to complete the voltage divider circuit that enables sensing of the gap state.

- **Mode 2: Training the motion control device to reproduce gestures**

  For training the motion control device, the following functionalities are added: The Arduino receives the recognized target gestures via USB, and senses the gap states during training mode, combining these inputs to generate the signals required to reproduce these gestures in the hand. The Arduino passes both target and current gestures to the display to visualize progress. During training, power for the ActuatorHeater is provided by the Arduino's 3.3V line. This is insufficient to trigger the actuation, but sufficient to detect the gap state and perform the training. For testing, the ActuatorHeater is powered externally and the gap sensor pins are automatically disconnected by MOSFETs to prevent overvoltage damage to the Arduino. This further illustrates that during testing no sensor input or processing is required to replicate the trained gestures.

Finally, all connections to the Artificial Hand are made via an 18-pin FPC connector. For the single T-SPG only 6 of those connections are used.

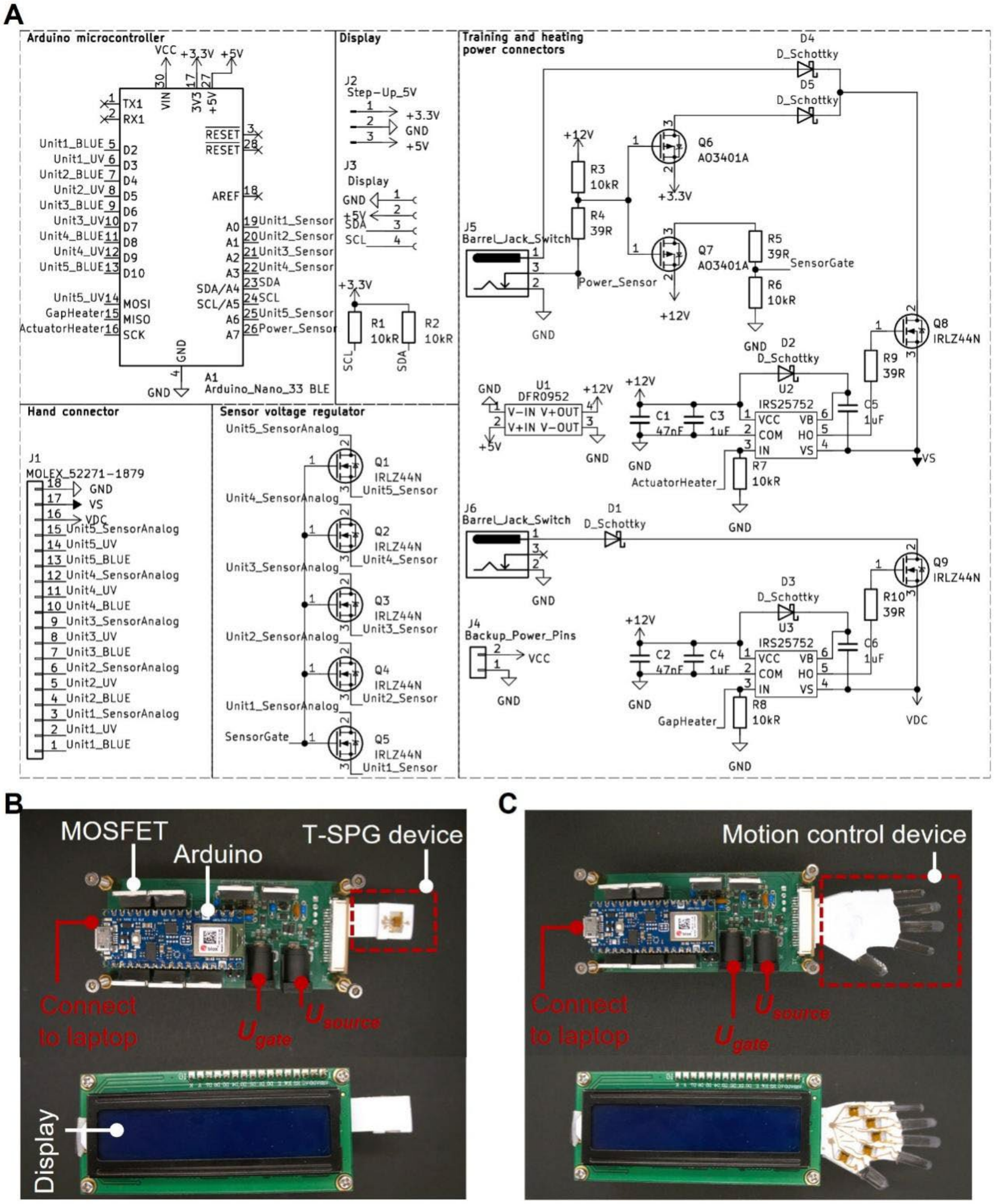

Fig. S8. Custom-built control system for training the T-SPG device and motion control device. **A**, The schematic of custom-built control system. **B**, Photograph of both sides of the physical control system interfaced with a single T-SPG device for the binary classification task. **C,** Photograph of both sides of the physical control system interfaced with the five-element motion control device for the gesture replication task.

Fig. S9. Input signal and corresponding thermal response during binary classification training.

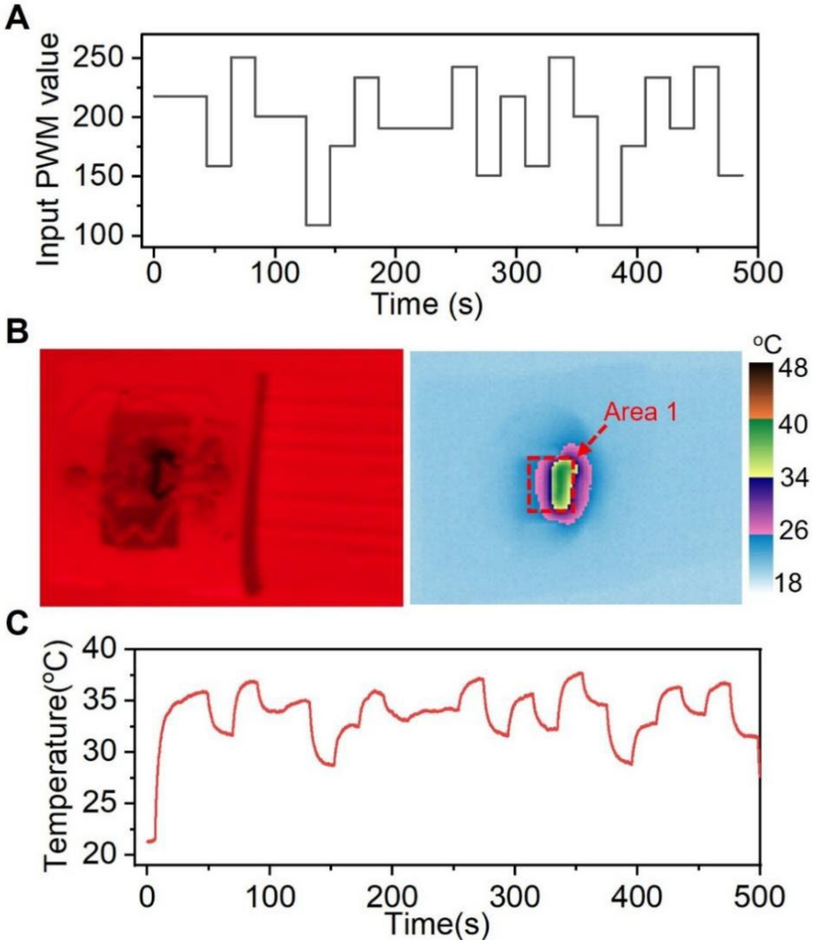

Fig. S9. Input signal and corresponding thermal response during binary classification training. **A**, Sequence of input Pulse-Width-Modulation (PWM) values over time, which are encoded from the training dataset of student grades. **B**, Optical (left) and thermal (right) images of the device during the application of the first data point (**G**=75). **C**, Temperature profile over time measured at "Area 1" in **B**, showing fluctuations corresponding to the PWM input in **A**.

Fig. S10. Unidirectional training of a single T-SPG for binary classification.

Before training, the T-SPG device was pre-conditioned with a strong UV light pulse (10 mW/cm² for 10 min) to ensure its initial $\boldsymbol{P_{th}}$ was set well below the target decision boundary defined by the dataset. The same training dataset and conditions were then applied to this T-SPG device. As shown in Fig. S10, the system now exclusively uses blue light to correct errors by raising $\boldsymbol{P_{th}}$**.** This result

confirms that the training process of the T-SPG is unidirectional, with the required optical modulation (either UV or blue light in principle) being determined by the initial state of the material's memory.

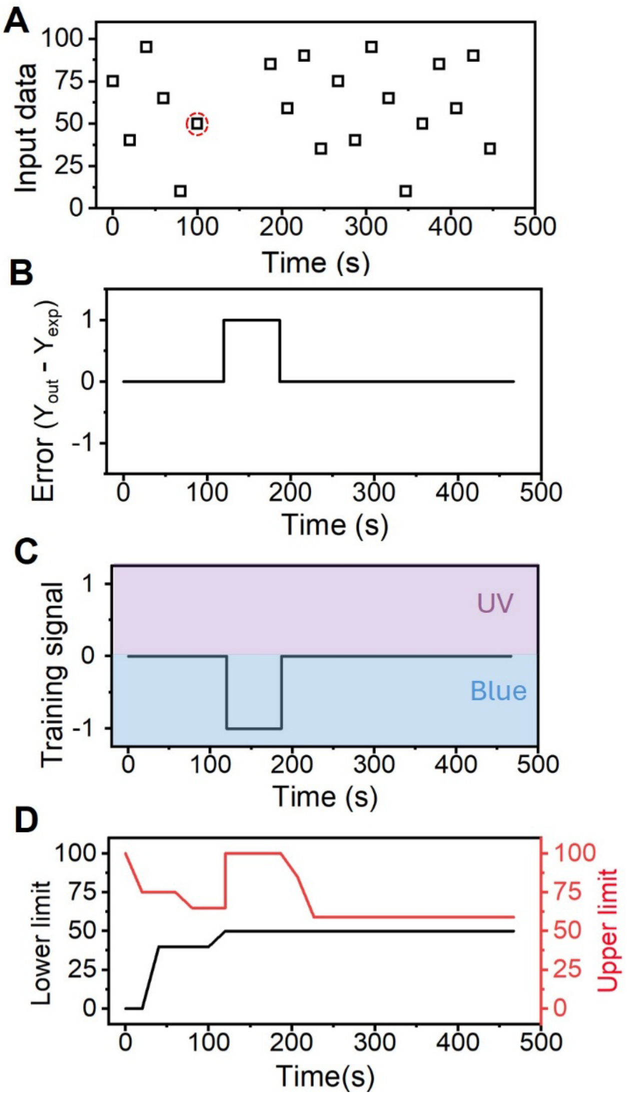

Fig. S10. Unidirectional training of a single T-SPG for binary classification. A, Sequence of input student grades from the training dataset over time. The red circles indicate data points that triggered optical correction. **B**, Output error ($Y_{out}-Y_{exp}$) for each data point during optimizing. **C**, Corresponding optical modulation signal used to update the P_{th}. **D,** Evolution of the inferred passing score bounds over training. The upper (red) and lower (black) limit progressively converge to define the decision boundary.

Fig. S11. Actuation behaviour of a single LCON-based actuator.

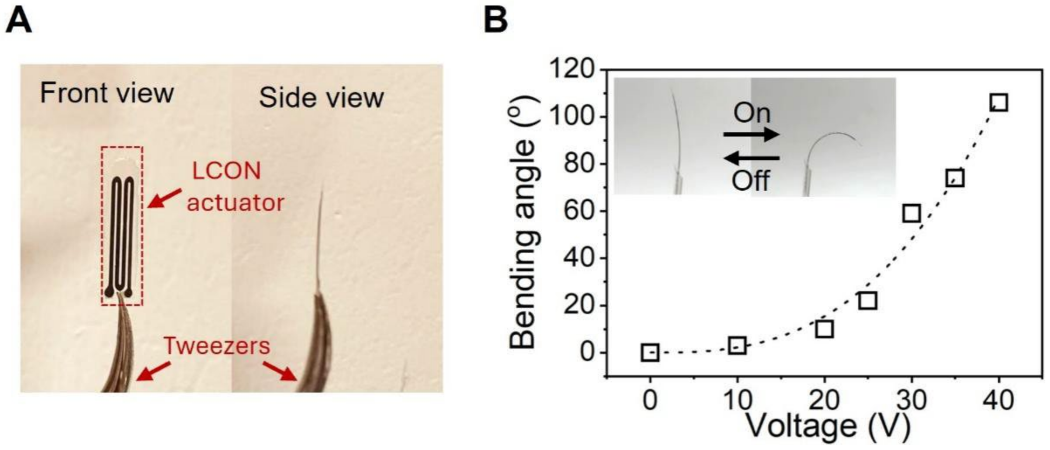

Fig. S11. Actuation behaviour of a single LCON-based actuator. A, Photographs of a single LCON actuator, showing the printed heating electrode (front view) and its profile (side view). **B**, Bending angle of the actuator as a function of the applied DC voltage. The heating electrode has a resistance of approximately 40 kΩ. Inserted photographs showing the actuator in its straight ('Off') and bent ('On') states. Upon applying voltage, the film bends toward the LCON side due to the contraction of the LCON.

Fig. S12. Layer-by-layer print paths for the fabrication of the LCON-based artificial hand

The LCON hand was fabricated using a multi-step direct ink writing (DIW) procedure. The designed print paths are illustrated in Fig. S12. The process began with the preparation of a thick sacrificial layer by manually scraping a 10 wt% polyvinyl alcohol (PVA) solution onto a pre-cleaned microscope slide. The subsequent printing steps were performed layer-by-layer as follows:

1. **First Conductive Layer**: A conductive ink blend was printed and thermally cured (120 ºC for 20 min) to form the gating electrodes for the five T-SPG units and the heating electrodes for the five LCON actuators.
2. **Second Conductive Layer**: A different conductive ink (Elepaste NP1) was printed and thermally cured (90 ºC for 20 min) to form the five gap electrodes and their associated conductive tracks.
3. **Insulating Layers**: A stretchable insulating ink (SI3104) was printed and cured at 120 °C for 10 minutes. This step was repeated one time to ensure sufficient electrical insulation.
4. **Final Conductive Layer**: Elepaste NP1 was printed and thermally cured again to complete the two-layer circuit architecture.
5. **Finger LCON Actuators**: Pure liquid crystal oligomer was printed in the finger regions and then photo-crosslinked at a low temperature (8 °C) for 20 minutes using UV light source (Omniture S2000, 300–500 nm, 30 mW/cm²). This low-temperature curing step was critical to prevent undesired curling and maintain finger straightness after the completed device was detached from the substrate.
6. **T-SPG Regions**: The azobenzene-functionalized (10 mol%) LCON was printed over the T-SPG areas and photo-crosslinked under a nitrogen environment using a 565 nm green LED for 1 hour, typically at 28 °C.
7. **Hand Body**: The remaining regions of the artificial hand were printed with pure liquid crystal oligomer and photo-crosslinked using UV light source (Omniture S2000, 300–500 nm, 30 mW/cm²), equipped with a high-pass optical filter ($\lambda > 400$ nm), for 20 minutes under a nitrogen atmosphere, typically at 25 °C.

Following printing and curing, the samples were immersed in water overnight to dissolve the PVA sacrificial layer, and then carefully peeled off from the substrate. The films were air-dried for over 24 hours to remove residual water. Subsequently, a handmade die was used to cut through the gap

electrodes and the LCON film to define the initial micro-gaps in all five T-SPG units. This step was performed at an elevated temperature (typically 45 °C), allowing the gaps to expand upon cooling to room temperature due to the thermal expansion of the LCON.

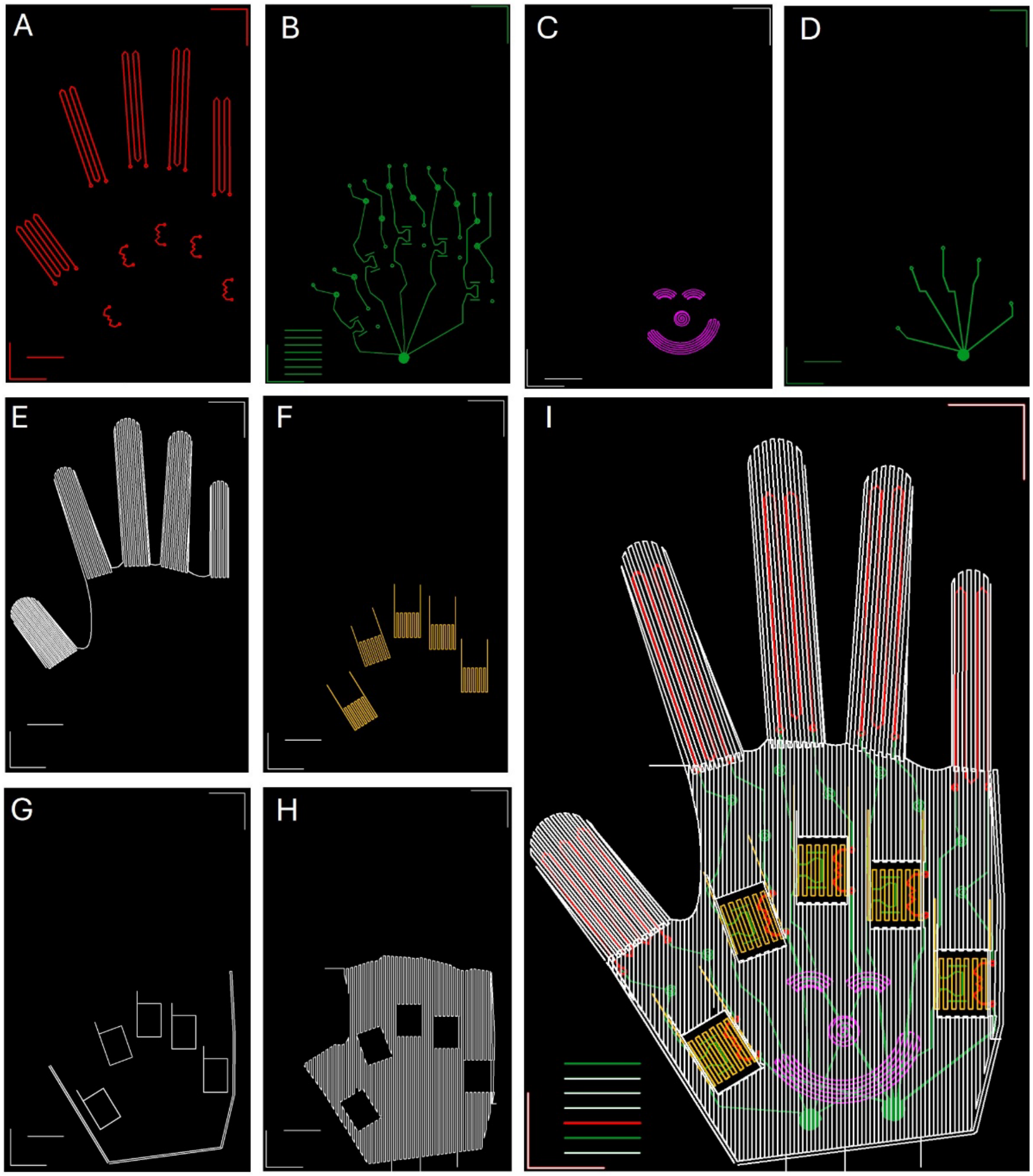

Fig. S12. Layer-by-layer print paths for the fabrication of the LCON-based artificial hand. A, Heating electrodes for the LCON fingers and the gating electrodes for the T-SPGs. **B,** Gap electrodes and the first layer of conductive tracks. **C,** Insulating layer made from a stretchable ink. **D,** Final conductive tracks to complete the two-layer circuit. **E,** Actuatable fingers, made of pure LCON. **F,** Azo-LCON in the T-SPG regions. **G,** Border of the hand, made of pure LCON. **H,** Palm of the hand, surrounding the T-SPG regions, made of pure LCON. **I,** Overlay of all print paths showing the complete design of the artificial LCON hand.

Fig. S13. Assembly process and circuit layout of the F-PCB for the motion control device

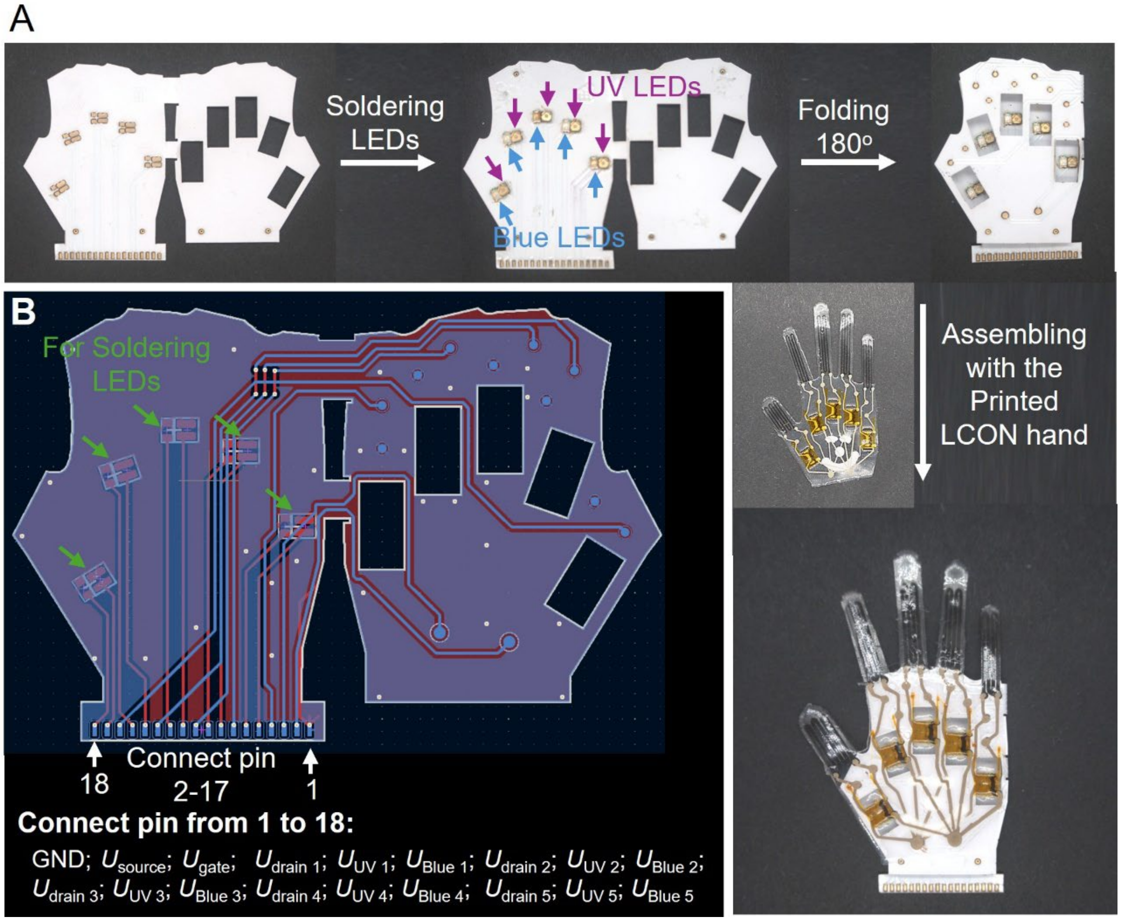

Fig. S13. Assembly process and circuit layout of the F-PCB for the motion control device. A, Step-by-step assembly sequence of the motion control device. Five UV LEDs and five blue LEDs, indicated by purple and blue arrows respectively, are soldered onto to initial flat F-PCB. The F-PCB is subsequently folded 180°, after which the printed LCON hand is assembled onto it to yield the motion control device. **B**, Circuit layout of the double-layer F-PCB. Red and blue tracks represent the top and bottom circuit layers, respectively. Green arrows indicate the soldering pads for the LEDs. The detailed pinout for the 18-pin connector is defined below the layout, specifying the terminals for ground (GND), actuator power ($\boldsymbol{U}_{source}$), T-SPG gating ($\boldsymbol{U}_{gate}$), gap state monitoring ($\boldsymbol{U}_{drain}$), and individual LED control.

Fig. S14. Vision-based recognition of Chinese number gestures

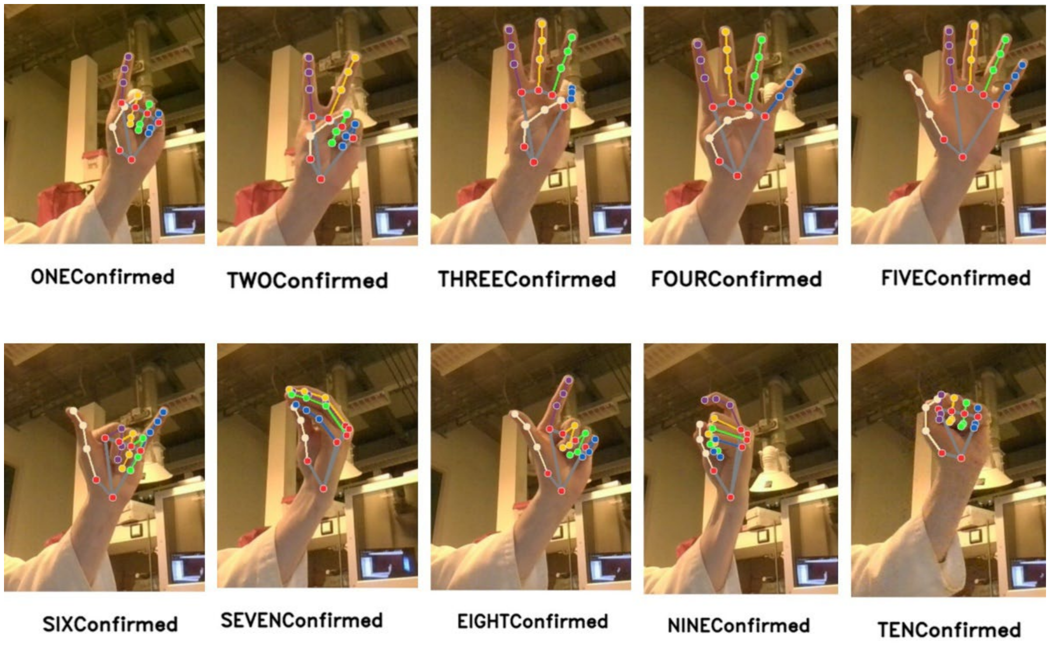

Fig. S14. Vision-based recognition of Chinese number gestures. Snapshots from the real-time gesture recognition system, showing the successful identification of Chinese number gestures from "1" to "10". The digital overlay of hand landmarks are generated using the MediaPipe library, while overall image processing is handled by OpenCV in Python. Landmarks are converted to gestures by a custom-trained MediaPipe machine-learning model. This vision system provides the target input to the controller for training the motion control device

Fig. S15. Thermal stabilization of the motion control device before activating optical modulation.

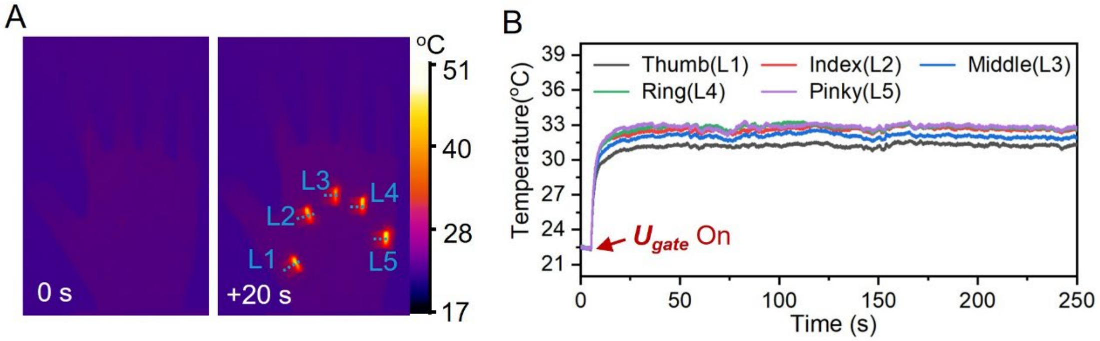

Fig. S15. **Thermal stabilization of the motion control device before activating optical modulation. A**, The thermal images of the motion control device before and after applying $\boldsymbol{U_{gate}}$ (5.0 V) for 20s. The labels L1-L5 indicate the temperature monitoring positions on each of the five T-SPGs (Thumb to Pinky). **B**, Temperature profiles for each of the five T-SPG units during the initial stabilization period. The plot shows that all five units reach and maintain a stable and uniform temperature of approximately 31 °C within 20 s, ensuring a consistent starting condition for the training protocol.

Fig. S16. Training the motion control device to reproduce the Chinese number gesture "1"

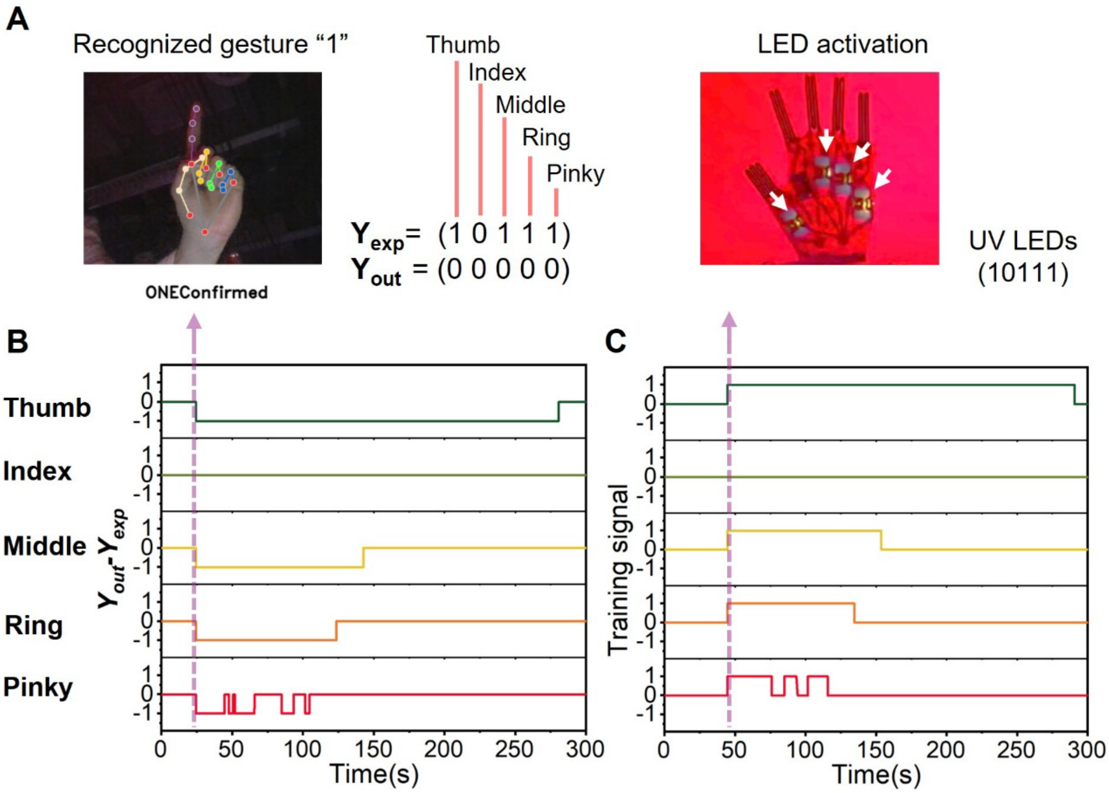

Fig. S16. Training the motion control device to reproduce the Chinese number gesture "1". **A**, Snapshots illustrating the key stages of the vision-based training loop. A human hand presents the target gesture "1", which is recognized by the vision system. The recognized gesture is encoded into a target output vector ($\mathbf{Y_{exp}}$). The controller monitors the actual output vector ($\mathbf{Y_{out}}$) and activates the corresponding LEDs (indicated by white arrows) on the motion control device to optimize P_{th}. **B**, The detected output error from each of the five T-SPG channels. **C**, Corresponding optical training signals for each channel.

Fig. S17. The chemical structure of components of the pure-LCON.

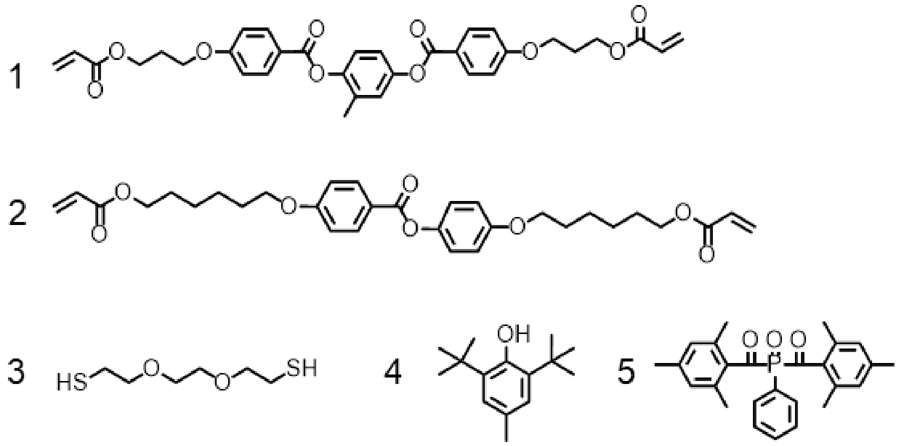

Captions for the supplementary movie files

Movie S1: Optically driven actuation behavior of the T-SPG. The video shows the gap closing upon UV irradiation (12.5 mW/cm²). After the UV is turned off, the gap partially reopens over time. Approximately 2 minutes later, blue light (1.8 mW/cm²) is applied, which further accelerates the reopening of the gap.

Movie S2: Electrically driven actuation behavior of the T-SPG. The video demonstrates gap narrowing when an electrical signal (U_{gate}) with a power of 17.5 mW is applied. The gap width decreases due to Joule heating-induced contraction of the Azo-LCON.

Movie S3: The training process of a single T-SPG for the binary classification task. The video shows the training loop using a custom Arduino-based control system. The system applies UV or blue light based on classification errors. The device temperature is monitored via an infrared camera. The iterative process adjusts the threshold power (P_{th}) until the target classification accuracy is achieved, as described in Fig. 2C and 2D.

Movie S4: The layer-by-layer DIW fabrication process of the artificial LCON hand. The video illustrates the sequential printing of heating/gating electrodes, connecting tracks, insulating layers, and LCON materials (pure LCON and Azo-LCON) to build the five-finger hand structure.

Movie S5: The training process of the motion control device for gesture replication. The video shows the closed-loop training of the motion control device to reproduce the Chinese number gesture “1”. A Python-based vision system (MediaPipe + OpenCV) recognizes the target gesture in real time, while an infrared camera monitors the device temperature. Based on the recognition result, the system activates the corresponding UV/blue LEDs to adjust the threshold power (P_{th}) of the required fingers (thumb, middle, ring, pinky).

Movie S6: Demonstration of the trained motion control device reproducing human gestures. The video shows the trained motion control device independently reproducing Chinese number gestures “1” to “6”, “8”, and “10” under input electric signals (U_{gate} = 5 V, U_{source} = 40 V), with no external control system. Each gesture corresponds to the intended bending pattern of the five fingers, confirming successful training and actuation.
